# Energy stress modulation of AMPK/FoxO3 signaling inhibits mitochondria-associated ferroptosis

**DOI:** 10.1016/j.redox.2023.102760

**Published:** 2023-05-24

**Authors:** Sufang Zhong, Wenjin Chen, Bocheng Wang, Chao Gao, Xiamin Liu, Yonggui Song, Hui Qi, Hongbing Liu, Tao Wu, Rikang Wang, Baodong Chen

**Affiliations:** a Department of Neurosurgery, Peking University Shenzhen Hospital, Shenzhen, China; b Jiangxi University of Traditional Chinese Medicine, Nanchang, China; c Key Laboratory of Evaluation of Traditional Chinese Medicine Efficacy (Prevention and Treatment of Brain Disease with Mental Disorders); Key Laboratory of Depression Animal Model Based on TCM Syndrome, Jiangxi Administration of Traditional Chinese Medicine; Key Laboratory of TCM for Prevention and Treatment of Brain Diseases with Cognitive Dysfunction, Jiangxi University of Chinese Medicine, Nanchang, China

**Keywords:** AMPK, FoxO3a, Energy stress, Ferroptosis, Lipid peroxidation

## Abstract

Cancer cells and ischemic diseases exhibit unique metabolic responses and adaptations to energy stress. Forkhead box O 3a (FoxO3a) is a transcription factor that plays an important role in cell metabolism, mitochondrial dysfunction and oxidative stress response. Although the AMP-activated protein kinase (AMPK)/FoxO3a signaling pathway plays a pivotal role in maintaining energy homeostasis under conditions of energy stress, the role of AMPK/FoxO3a signaling in mitochondria-associated ferroptosis has not yet been fully elucidated. We show that glucose starvation induced AMPK/FoxO3a activation and inhibited ferroptosis induced by erastin. Inhibition of AMPK or loss of FoxO3a in cancer cells under the glucose starvation condition can sensitize these cells to ferroptosis. Glucose deprivation inhibited mitochondria-related gene expression, reduced mitochondrial DNA(mtDNA) copy number, decreased expression of mitochondrial proteins and lowered the levels of respiratory complexes by inducing FoxO3a. Loss of FoxO3a promoted mitochondrial membrane potential hyperpolarization, oxygen consumption, lipid peroxide accumulation and abolished the protective effects of energy stress on ferroptosis in vitro. In addition, we identified a FDA-approved antipsychotic agent, the potent FoxO3a agonist trifluoperazine, which largely reduced ferroptosis-associated cerebral ischemia-reperfusion (CIR) injuries in rats through AMPK/FoxO3a/HIF-1α signaling and mitochondria-dependent mechanisms. We found that FoxO3a binds to the promoters of SLC7A11 and reduces CIR-mediated glutamate excitotoxicity through inhibiting the expression of SLC7A11. Collectively, these results suggest that energy stress modulation of AMPK/FoxO3a signaling regulates mitochondrial activity and alters the ferroptosis response. The regulation of FoxO3a by AMPK may play a crucial role in mitochondrial gene expression that controls energy balance and confers resistance to mitochondria-associated ferroptosis and CIR injuries.

## Highlights

• Energy stress induced AMPK/FoxO3a activation and inhibited ferroptosis induced by erastin.
• Energy stress inhibits mitochondria-related gene expression and regulates mitochondrial activity by inducing FoxO3a.
• FoxO3a agonist trifluoperazine reduced ferroptosis-associated cerebral ischemia/reperfusion(CIR) injury in rats through AMPK/FoxO3a/HIF-1α signaling and mitochondria-dependent mechanisms.
• FoxO3a binds to the promoters of SLC7A11 and reduces CIR-mediated glutamate excitotoxicity through inhibiting the expression of SLC7A11.

## Graphical abstract

In the adaptation to hypoxia or low nutrient conditions, energy stress-mediated AMPK/FoxO3a signaling activation inhibited HIF-1α/SLC7A11. Glucose deprivation reduced MMP hyperpolarization, mitochondrial mass, OXPHOS complexs, oxygen consumption, lipid peroxidation and ATP levels, resulting in resistance to ferroptosis induced by erastin. TFP, a novel FoxO3a activator reduced ferroptosis-associated cerebral ischemia-reperfusion (CIR) injuries through repression of HIF-1α/SLC7A11 expression and mitochondrial activity.

**Figure:**
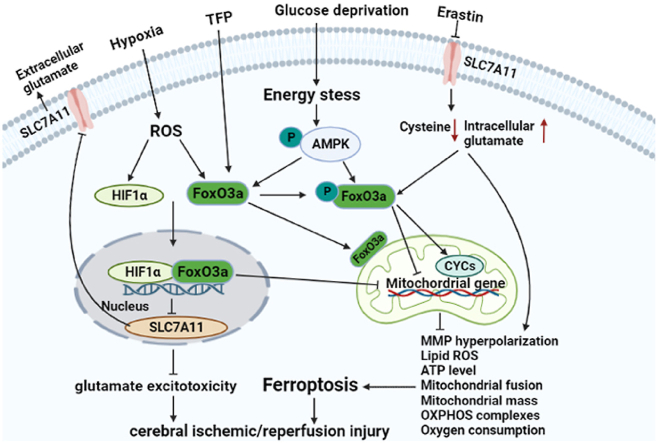

## 1 Introduction

Ferroptosis is a major mechanism for cell death associated with various human diseases, such as ischemia/reperfusion injury (IRI), brain damage, and cancer [[1], [2], [3]]. Ferroptosis is characterized by the accumulation of lipid peroxidation, especially those resulting from the oxidation of polyunsaturated fatty acids (PUFA) in membrane phospholipids. Mitochondria are the central hubs for cellular bioenergetics and are the most important source of ROS in mammalian cells, which is vital for regulating ferroptosis [4]. Mitochondria is a crucial player in erastin-induced or cysteine deprivation-induced ferroptosis, but not induced by RSL3 for inhibiting glutathione peroxidase-4 (GPX4) [4]. Ferroptosis is different with other forms of cell death because of dramatic morphological changes in mitochondria, including cristae enlargement and mitochondrial fragmentation; mitochondrial fragmentation and accumulation around the nucleus are increased in response to erastin toxicity [5].The role of mitochondrial morphology and function in ferroptosis are discussed heatedly.

The maintenance of cellular energy homeostasis is pivotal for organismal homeostasis throughout life. Glucose deprivation induces energy stress, resulting in adaptive responses to restore energy homeostasis. The AMP-activated protein kinase (AMPK) plays an important role in cellular and systemic energy homeostasis. Energy stress increased the phosphorylation of FoxO3a and acetyl-CoA carboxylase (ACC), two known AMPK substrates, and AMPK-mediated ACC phosphorylation inhibited fatty acid synthesis and was resistant to ferroptosis under glucose starvation conditions [6]. Whether energy stress mediated AMPK/FoxO3a signaling regulates ferroptosis remains largely unknown.

AMPK promotes FoxO3a transcriptional functions and plays an important role in regulating mitochondrial homeostasis [7]. FoxO3a activation inhibits mitochondrial gene expression through down-regulation of c-Myc function and alters the hypoxia response [8]. FoxO3a is induced in hypoxia and can prevent hypoxia-inducible factor 1α (HIF-1α)-dependent gene expression by directly binding to HIF-1α [9]. In particular, mitochondria derived ROS are required for HIF-1α induction in hypoxia [10]; FoxO3a activation prevented an increase in ROS levels in hypoxic cells by decreasing HIF-1α accumulation [11]. HIF-1α promotes cystine-glutamate antiporter (SLC7A11/xCT) expression which contributes to cerebral ischemia-reperfusion (CIR)-mediated glutamate release and excitotoxicity [12]. However, inhibition of SLC7A11 activity by erastin can deprive of cellular cysteine and GSH and disrupt cellular redox homeostasis, resulting in ferroptosis [13]. Thus, the functional relevance of HIF1α/SLC7A11 and mitochondria-associated ferroptosis in CIR is still highly debatable.

In this study, we would like to investigate the relationship between AMPK/FoxO3a activity and mitochondria function in ferroptosis under energy stress. Our findings suggest that energy-stress-mediated AMPK/FoxO3a signaling activation inhibits ferroptosis via mitochondria-dependent mechanisms. We identified the FoxO3a activator trifluoperazine (TFP), a FDA-approved antipsychotic agent that reduced ferroptosis-associated brain ischemia/reperfusion (CIR) injury in rats through AMPK/FoxO3a/HIF-1α/SLC7A11 signaling and mitochondria-dependent mechanisms.

## 2 Materials and methods

### 2.1 Animals

Male Sprague-Dawley (SD) rats (SPF grade, weighing 250–280 g) were purchased from the Nanjing Coris Biotechnology Co. (Nanjing, China). The rats were housed in an environment with standard lighting conditions (12 h light/dark cycle), controlled temperature (21–25 °C) and humidity (40–60%), and with freely accessible food and water. All animal experiments were approved by the Ethics Committee of Jiangxi University of Chinese Medicine.

### 2.2 Cell line,primary cells and cell transfection

MCF-7 cells, HEK293T cells and BV-2 cells were purchased from Shanghai Cell Bank of Chinese Academy of Sciences. Primary mouse embryonic fibroblasts (MEFs) were established from embryos at E13.5 as previously described [14]. All cell lines and MEFs were cultured in Dulbecco's modified Eagle's medium containing 10% fetal bovine serum and 1% (v/v) penicillin/streptomycin at 37 °C incubator with humidified atmosphere of 5% CO2.

The small interfering RNA (siRNA) of FoxO3a was synthesized by GenPharma Co., Ltd. (Shanghai, China), and the sequences were as follows: FoxO3a sense: 5ʹ-GGAACGUGAUGCUUCGCAATT-3ʹ, antisense: 5ʹ-UUGCGAAGCAUCACGUUCCTT-3ʹ. The BV-2 cells were transfected with siRNA of FoxO3a or negative control siRNA using a lipofectamine 3000 reagent according to the manufacturer's protocols. A lentivirus(Santa Cruz Biotechnology,sc-37887-V) was transduced to knock down FoxO3a in MCF-7 cells. Cells were transduced with lentivirus (multiplicity of infection = 50). Day 5–6 and forward, select stable clones expressing the shRNA via Puromycin dihydrochloride (Santa Cruz Biotechnology, sc-108071) selection. Replace the medium with fresh puromycin-containing medium every 3–4 days, until resistant colonies can be identified. Pick several colonies, expand them and assay them for stable shRNA expression by Western blot.

### 2.3 Cell viability assay

CCK-8 kit (Sigma, 969920) was used to measure cell viability. HEK293T, MCF-7 and MEFs cells in logarithmic growth were inoculated into 96-well plates at a density of 3 × 10^4^/well. The old medium was removed the next day, replaced with high-sugar medium and sugar-free medium, and treated for 24 h with the same gradient concentration of erastin. Following treatment, CCK-8 reagent of 10ul (100μl culture base/well) was added to each well. Then incubate for 1 h in a 37 °C, 5% CO_2_ incubator. The absorbance at a wavelength of 450 nm was determined a using a microplate reader.

### 2.4 Western blot analysis

Cells and brain tissue were lysed in ice-cold RIPA buffer containing protease inhibitor, the same amounts of protein were separated using SDS-PAGE gel and transferred on PVDF membranes. The membranes were blocked in 5% skim-milk and then incubated overnight at 4 °C with the primary antibodies for mouse AMPK (1:1000,Proteintech,#66536-1-Ig), rabbit *p*-AMPK(Thr172) (1:1500,Cell Signaling technology,#2535), rabbit FoxO3a (1:1000,proteintech,#66428-1-Ig), rabbit *p*-FoxO3a(Ser413)(1:1000,Cell Signaling technology,#8174), rabbit CYCs (1:1000,Cell Signaling technology,#11940S), rabbit HMOX1 (1:1000,ABclonal,#A19062), rabbit SOD2 (1:1000,proteintech,#6647-1-Ig),mouse PGC1α(1:1000,proteintech,#66369-1-Ig),mouse TOM20(1:1000,proteintech,#66777-1-Ig),rabbit GPX4 (1:1000,ABclonal,#A11243),rabbit SLC7A11 (1:1000,ABclonal,#A2413),rabbit DRP1 (1:1500,proteintech,#12957-1-AP),mouse Hsp60 (1:1000,proteintech,#66041-1-Ig), rabbit *p*-MFF (ser172/ser146)(1:1500,Cell Signaling technology,#AF2365),mouse Total OXPHOS(1:2500,Abcam,#ab110413),mouse β-actin (1:1000,TRANS,#HC201).

### 2.5 Quantitative real-time PCR(qRT-PCR)

The total RNA was extracted using TRIzon Reagent (CWBIO, Beijing, China) according to the manufacturer's protocols. Reverse transcriptase reactions were performed using the First-Strand cDNA Synthesis Kit (Thermo Scientific, USA). The real-time PCR was performed with SYBR Green PCR Master Mix (Yeasen Biotech Co., Ltd., Shanghai, China) under the following conditions: 95 °C for 5 min, followed by 40 cycles of 95 °C for 10s, 60 °C for 30 s. Mitochondrial DNA (mtDNA) copy number was determined by quantitative real-time PCR as described [8,15], quantification of mtDNA was performed by use of the ratio of mitochondrial gene (*cytochrome b*) to nuclear gene (*β-actin*).Other Genes expression levels were normalized to the signals of GADPH expression. The primers were listed in Table S1.

### 2.6 Intracellular ATP level measurement

Intracellular ATP levels were detected by ATP Assay Kit (Bryotime, S0026) according to the manufacturer's instructions. MCF-7 cells were seeded in 6-well culture plates. The next day, the medium was changed to a high-sugar and sugar-free medium, and erastin was added at a concentration of 16 μM for 24 h. Discard the medium and wash three times with PBS. Add 200ul of ATP detection lysis buffer (S0026-3) to each well to lyse cells. Then the luminescence of each well was subsequently measured with a microplate reader.

### 2.7 Determination of lipid peroxidation

MCF-7 cells were seeded in 24 wells with cell slides. The next day, the medium was changed to a high-sugar and sugar-free medium, and erastin was added at a concentration of 16 μM for 24 h. Discard the medium and wash three times with PBS. Then C11-BODIPY581/591 (Invitogen) at a concentration of 10 μM was added and incubated in the dark for 30 min. Continue to wash 3 times with PBS. Cells were fixed with 4% paraformaldehyde and observed with a silver light microscope at 400 × magnification.

Cells were seeded at a density of 2.0 × 10^5^ per well on coverslips placed in a 6-well dish. The next day, the medium was changed to a high-sugar and/or sugar-free medium, and erastin was added at a concentration of 16 μM for 24 h. Coverslips were washed in HBSS and incubated in HBSS containing 10 μM BODIPY 581/591C11 (Invitrogen) and 200 nM MitoTracker Deep Red FM (Invitrogen) for 20 min. Coverslips were then inverted onto microscope slides. Observed with a silver light microscope at 400 × magnification and images were processed in Photoshop (Adobe).

### 2.8 Mitochondrial membrane potential (MMP) measurement

The change in MMP was assessed using a JC-1 MMP Assay Kit (Solarbio Science&Technology, Beijing, China). Briefly, after incubating with the JC-1 staining work solution for 20 min at 37 °C, the MCF-7 cells were washed twice with HBSS and imaged using a fluorescence microscope. The red fluorescent aggregate indicates a healthy mitochondrion with normal membrane potential, whereas the green fluorescent monomer indicates loss of MMP.

### 2.9 Immunofluorescence

Rat brains were fixed with 4% polyformaldehyde for 48 h. They were then collected in a 30% sucrose solution for 48 h before being snap frozen and cryosectioned at 15 μm thickness. Sections were incubated in 0.5% Triton X-100 for 20 min and blocked with PBS containing 3% goat serum for 1 h at room temperature before permeating overnight at 4 °C with primary antibodies. After three times washes by PBS (10 min each), sections were incubated with respective secondary antibodies for 2 h, including goat anti-mouse IgG (AlexaFluor-488, 1:200,Abcam,#ab150117) and goat anti-rabbit IgG (AlexaFluor-594,1:200, Abcam,#ab150080). A fluorescent microscope was implemented of observation at 400× magnification. We used the following primary antibodies: mouse anti-FoxO3a (1:200,proteintech,#66428-1-Ig), rabbit anti-GPX4 (1:200,ABclonal,#A11243), rabbit anti-SLC7A11 (1:200,ABclonal,#A2413), rabbit anti-Hif1α (1:200,proteintech,#20960-1-AP）.

### 2.10 Whole-genome ChIP-seq

ChIP-seq was performed by Wuhan IGENEBOOK Biotechnology Co., Ltd (Wuhan, China). Briefly, BV-2 cells (2 × 10^7^) were crosslinked by formaldehyde (1%) at 26 °C for 10 min. Glycine (0.125 mol/L) was added to stop the crosslinking, and chromatin was sonicated into 200–500 bp fragments using sonication. The anti-FoxO3a antibody (PA1-805, Thermo Fisher Scientific Co., Ltd, USA) or IgG (ab231712, Abcam Co., Ltd, Shanghai, China) antibody and beads were applied to pull down the target protein, and proteinase K (345 μg/mL) (1245680100, Merck Co., Ltd, Germany) was used to digest proteins at 45 °C overnight. Immunoprecipitated DNA was used to construct sequencing libraries following the protocol provided by the I NEXTFLEX® ChIP-Seq Library Prep Kit for Illumina® Sequencing (NOVA-5143-02，Bio Scientific, USA) and sequenced on Illumina Xten with the PE 150 method.

### 2.11 Immunohistochemistry assay

Rat brains were fixed with 4% polyformaldehyde, then processed into 5 μm-thick sections and immunostained with specific antibodies for 4-HNE(1:100,Bioss antibdies,#bs-6313R). The slides were imaged under a light microscope (Olympus BX53). The percentage of positive cells was calculated by counting under high magnification ( × 400).

### 2.12 Rat models of transient middle cerebral artery occlusion (MCAO)

The rats were anesthetized with isoflurane gas (5% induction and 2.5% maintenance), and then cut off the neck skin, where they found the right common carotid artery (CCA), isolated and ligated the external carotid artery (ECA) and the internal carotid artery (ICA). A 0.36–0.37 mm monofilament nylon suture with a heat-blunted tip was introduced into the right CCA to the internal carotid artery (ICA) through a small incision in the CCA and then gently advanced from the CCA bifurcation to block the origin of the right middle cerebral artery. Reperfusion was allowed after 2 h of MCAO by monofilament removal. The body temperature of the rats was maintained during and after surgery until recovery from anesthesia. The suture remained there until the rats were sacrificed. Sham-operated rats underwent the same procedures except for the transient MCAO.

### 2.13 TTC staining

TTC staining was exploited to detect infarct volume. In brief, rats were deeply anesthetized with isoflurane gas and sacrificed by decapitation. The brain was rapidly removed and cut into six coronal brain slices with an approximate thickness of 2 mm. These sections were stained with 2% 2, 3, 5-triphenyltetrazolium chloride (Solarbio Science&Technology Co.Ltd, Beijing, China) for 30 min at 37 °C in the dark. The TTC was dissolved in physiological saline solution. Viable tissues are stained deep red while the infarcts remain unstained. Infarcted areas were measured using Image J.

### 2.14 Mitochondria isolation

All steps were carried out in accordance with the instructions of the Cell Mitochondria Isolation Kit (Beyotime, China). First, weigh 100 mg of brain tissue and wash it with cold PBS, then add pre-cooled Lysis and grind the tissue 20 times at 4 °C in an ice bath. Second, the cold lysis buffer was mixed with the tissue. The tissue homogenate was transferred to a clean centrifuge tube containing medium buffer, mixed gently, and centrifuged at 1200g for 5 min at 4 °C. The supernatants were transferred to new tubes and centrifuged at 4 °C 7000 g for 10 min. The precipitate containing mitochondria was lysed with mitochondrial lysis buffer for Western blot.

### 2.15 Glutamate assay

The intracellular glutamate concentration was measured by assay kit from olarbio Science&Technology (BC1585) according to the manufacturer's instructions, and the reaction product was measured by a microplate reader at 340 nm.

### 2.16 MDA and Fe^2+^ detection

The MDA concentration in the penumbral brain tissue was measured by the thiobarbituric acid method using a MDA assay kit (BC0025, Solarbio Science&Technology, Beijing, China). MDA was calculated based on cellular protein concentration and expressed as nmol of MDA per gram of protein (nmol/g). Furthermore, the Fe^2+^ level was measured using kits from olarbio Science&Technology (593 nm) according to the manufacturer's instructions.

### 2.17 OCR and ECAR measurements

The oxygen consumption rate (OCR) and extracellular acidification rate (ECAR) was measured by an XFp extracellular analyzer (Agilent Technologies, Santa Clara, CA, USA). MCF-7 transfected with FoxO3a shRNA were seeded at 3.0 × 10^4^ cells/well density in 8-well plates for overnight incubation to allow adherence to the plate. Then the cells were treated with erastin (16 μM) in glucose depletion medium for 6 h. After 6 h of erastin administration, the cells replaced to Seahorse XF assay Medium (Agilent, Santa Clara, CA, USA) pH 7.4 supplemented with 10 mM glucose, 1 mM pyruvate, and 2 mM glutamine. After monitoring 18 min of basal respiration, erastin (16 μM) was added to the system, then sequential injection 1 μM of mitochondrial inhibitors oligomycin, FCCP, and antimycin (AA) plus rotenone (AR) provided by the manufacturer (#101706–100, Agilent Technologies) every 20 min after monitoring. OCR and ECAR was automatically calculated using the Seahorse XFp software. Every point represents an average of three different wells.

### 2.18 Statistical analyses

All data were expressed as means ± SEM. The data significance was evaluated by PASW Software (SPSS Inc., Chicago, IL, USA). Statistical significance among various groups was calculated by one-way ANOVA using post-hoc tests, *p* *<* 0.05 was considered statistically significant.

## 3 Result

### 3.1 Energy stress inhibits erastin and RSL3-induced ferroptosis

**Fig. 1:**
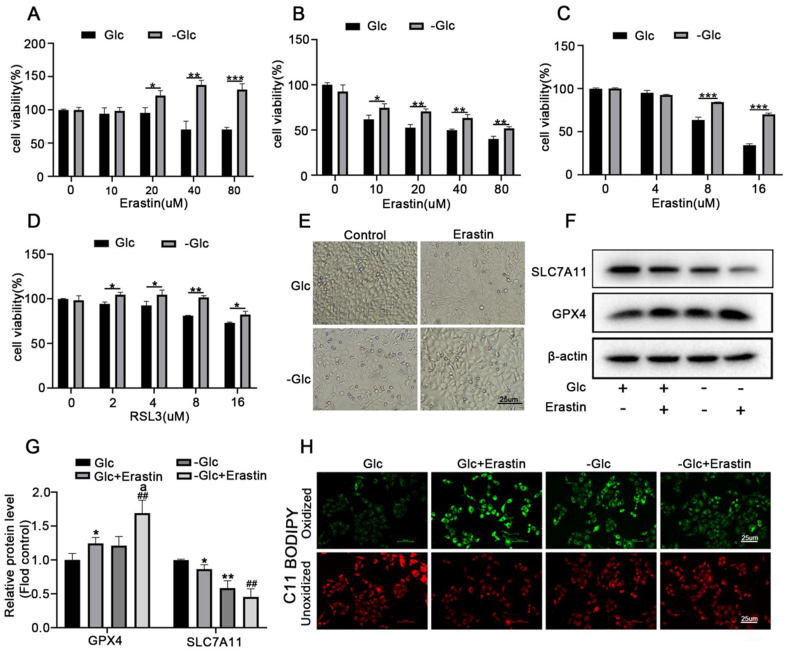
Energy stress inhibits ferroptosis. Cells were treated with different doses of erastin or RSL3 in normal and glucose depletion medium for 24 h then the cell viability of **(A)**HEK293T, **(B)**MEFs and **(C/D)**MCF-7 cells were measured by CCK-8 kit. **(E)** Representative images showing the cell morphological change in MCF-7 cells. **(F)** MCF-7 cells were treated with erastin(16 μM) in normal and sugar-free medium for 24 h, the protein level of GPX4 and SLC7A11 was determined by western-blot assay, respectively. **(G)**The quantitative analyses of GPX4 and SLC7A11 protein band intensities after β-actin normalization. **(H)** MCF-7 cells were treated as indicated for 24 h, the accumulation of lipid ROS was assessed by C11-BODIPY581/591 staining. *p < 0.05, **p < 0.01, ***p < 0.001 versus the Glc group; ^#^*p* < 0.05, ^##^*p* < 0.01 versus the -Glc group; ^a^*p*＜0.01 versus the Glc + erastin group. Data are expressed as mean ± SEM, n = 3.

To study the role of glucose deprivation in ferroptosis, we first investigated the effect of glucose deprivation on erastin- or RSL3-induced ferroptosis in HEK293T, mouse embryonic fibroblasts (MEFs) cells and MCF-7 cells. Cells were treated with different doses of erastin or RSL3 in normal and glucose depletion medium for 24 h. Consistent with previous findings, we found that erastin or RSL3 does dependent induced ferroptotic cell death, whereas glucose deficiency largely reversed erastin and RSL3-induced ferroptosis (Fig. 1A-B-C-D). We further explored the effect of glucose depletion on erastin-induced morphological change in MCF-7 breast cancer cells, and representative images showed that glucose deprivation reduced erastin-induced cell death (Fig. 1E). Glutathione peroxidase-4 (GPX4) and SLC7A11 is a central regulator of ferroptosis. Therefore, we used Western blot to further determine the expression of GPX4 and SLC7A11 in MCF-7 cells. The results showed that lipid peroxidation and oxidative stress were suppressed along with the up-regulation of the ferroptosis antagonism marker GPX4 while down-regulation of SLC7A11 under glucose deprivation conditions (Fig. 1F–G).Since the accumulation of lipid peroxidation is a hallmark of ferroptosis, we used C11-BODIPY581/591 probes to detect lipid peroxidation; the green fluorescence indicated oxidation type and the red fluorescence indicated non-oxidation type. As shown in Fig. 1H, erastin induced ROS accumulation, whereas treatment of glucose-deprived cells with erastin reduced lipid peroxidation.

### 3.2 Energy stress inhibits erastin-induced ferroptosis partly through the AMPK/FoxO3a signaling pathway

**Fig. 2:**
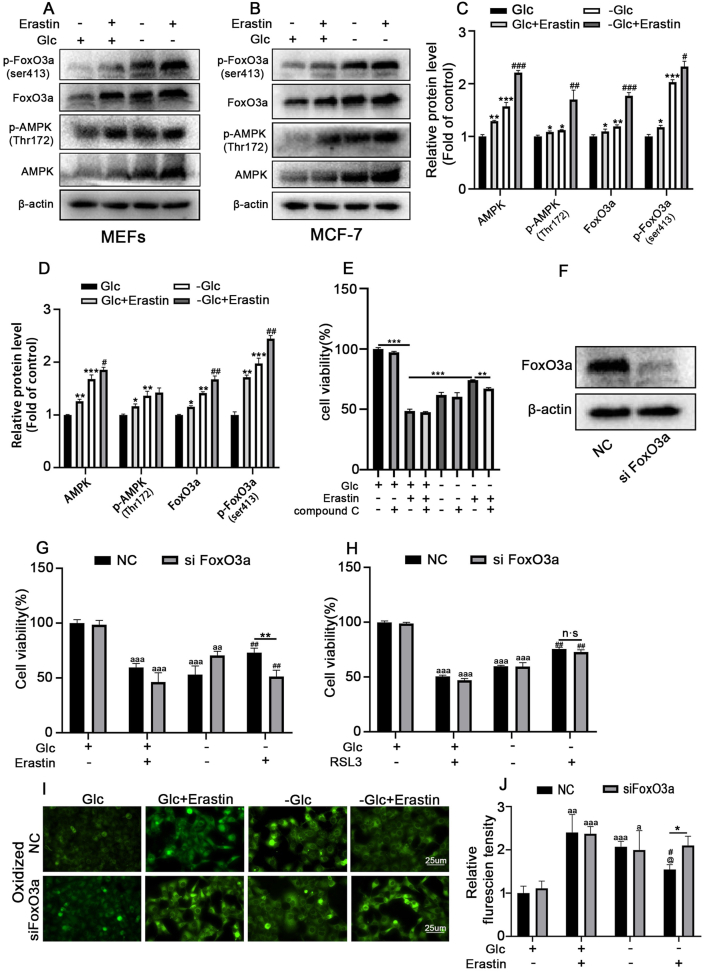
**Energy stress inhibits ferroptosis partly through AMPK/FoxO3a signaling pathway.** Cells treated with or without erastin(16 μM) and cultured in normal or sugar-free medium for 24 h Then the protein levels of FoxO3a, *p*-FoxO3a(Ser413), AMPK and *p*-AMPK(Thr172) in (**A/C**)MEFs and (**B/D**)MCF-7 cells were determined by Western blot. *p < 0.05, **p < 0.01, ***p < 0.0001 versus the Glc group. ^#^*p* < 0.05, ^##^*p* < 0.01, ^###^*p* < 0.001 versus the -Glc group. (**E)** MCF-7 cells were treated with compound C (2 μM) or erastin (16 μM) in normal and sugar-free medium for 36 h, then the cell viability was measured by CCK-8 kit. *p < 0.05, **p < 0.01, ***p < 0.001. **(F)** MCF-7 cells were transfected with FoxO3 specific siRNA for 24 h, the efficiency of knockdown was detected by western blotting analysis. 24 h after transfection with FoxO3a shRNA or Negative control (NC), MCF-7 cells were treated with 16 μM erastin **(G)** or 5 μM RSL3 **(H)** and cultured in normal or sugar-free medium for 24 h. Cell viability was measured by CCK-8 kit. **p < 0.01 versus NC group cultured in sugar-free medium, ^##^*p* < 0.01 versus the group cultured in sugar-free medium for **(G)** while ^##^*p* < 0.01 versus the erastin group cultured in normal medium for**(H)**，^aa^*p* < 0.01, ^aaa^*p* < 0.001 versus the control group. **(I/J)** 24 h after transfection with lentivirus containing FoxO3a shRNA, MCF-7 cells were treated with erastin and cultured in normal or sugar-free medium for 24 h. Oxidized C11-BODIPY581/591 (Green) indicating lipid ROS were imaged by fluorescent microscope.*p＜0.05,**p＜0.01 versus NC; ^aa^*p*＜0.01,^aaa^*p*＜0.001 versus the Glc group; ^#^*p*＜0.05，^##^*p*＜0.01 versus the erastin group;^@@^*p*＜0.01 versus the -Glc group. Data are expressed as mean ± SEM, n = 3.

AMPK/FoxO3a signaling play an important role in metabolic homeostasis under energy stress. To investigate the role of AMPK/FoxO3a signaling in energy-stress-mediated ferroptosis, MEFs and MCF-7 cells were treated with or without erastin under normal or glucose deprivation medium as indicated for 24 h and then performed a Western blot experiment. We found that MEF cells treated with erastin induced a significant increase in expression levels of AMPK and p- AMPK(Thr172), and this effect was further enhanced under the condition of glucose deprivation (Fig. 2A and C). We also found that the ratio between *p*-AMPK/p-FoxO3a and total protein of AMPK/FoxO3a was significantly increased during energy stress (Fig. S1). This finding was confirmed in MCF-7 breast cancer cells (Fig. 2B and D).

FoxO3a is a downstream target gene regulated by AMPK that controls energy balance and stress resistance in cells. AMPK phosphorylates FoxO3a at Serine 413 during glucose deprivation. Therefore, to further investigate whether AMPK-mediated FoxO3a activation plays a role in ferroptosis inhibition by energy stress, Western blot was used to detect the protein levels of FoxO3a/p-FoxO3a (Ser413) under energy stress conditions. The results showed that the protein levels of FoxO3a/p-FoxO3a (Ser413) were significantly increased after the erastin treatment, and these effects were further enhanced under energy stress conditions (Fig. 2A–D).

Next, in order to investigate whether AMPK is involved in glucose deprivation -mediated ferroptosis inhibition, MCF-7 cells cultured in normal or glucose-free medium were pretreated with compound C (an AMPK inhibitor), then the cells were induced to ferroptosis by erastin. We found that compound C significantly promoted ferroptosis induced by erastin under normal medium, glucose deprivation inhibited ferroptosis induced by erastin while compound C treatment reversed these effects (Fig. 2E). To further study whether FoxO3a plays any causal role in ferroptosis inhibition in the cancer cells. MCF-7 cells were transfected with FoxO3a shRNA to down-regulate FoxO3a. The efficiency of knockdown was confirmed by Western blot analysis. Knocking down FoxO3a in MCF-7 cells significantly restores ferroptosis sensitivity induced by erastin but not RSL3 (Fig. 2F, G, 2H). We further monitored lipid ROS accumulation using C11 BODIPY 581/591.We found knockdown FoxO3a in erastin-treated cells reversed the decline level of lipid ROS accumulation under energy stress(Fig. 2I–J). Taken together, our findings suggested that FoxO3a is a critical downstream effector of AMPK in regulating ferroptosis inhibition under energy stress conditions.

### 3.3 FoxO3a activation induced by glucose depletion regulated the expression of antioxidative enzymes

**Fig. 3:**
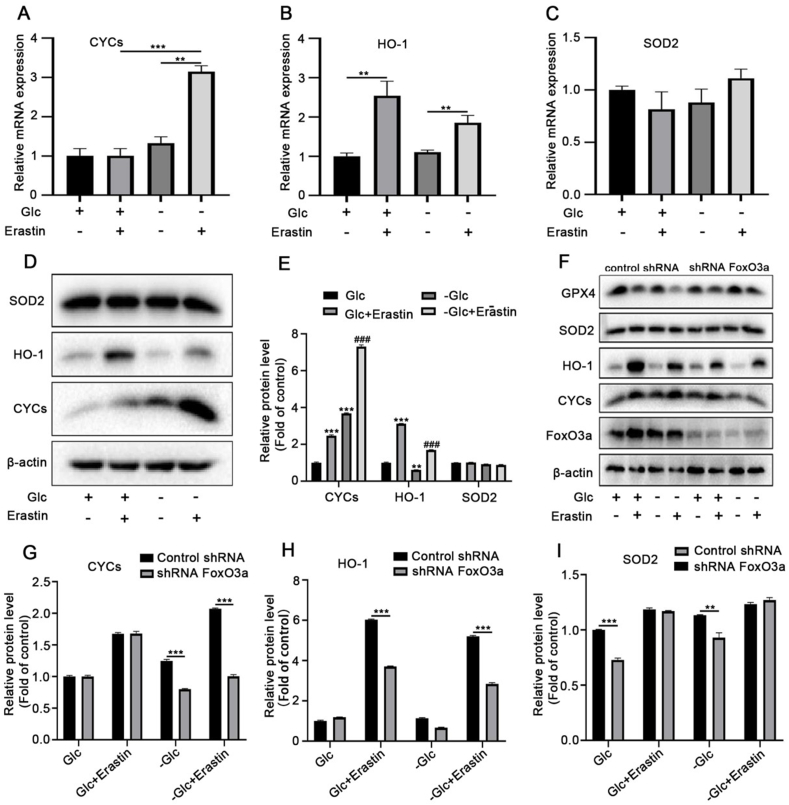
**FoxO3a activation induced by glucose depletion regulates the expression of anti-oxidative enzymes.** MCF-7 cells were treated with or without erastin(16 μM) and cultured in normal or sugar-free medium for 24 h. The mRNA levels of CYCs (**A**), HO-1 (**B**) and SOD2 (**C**) were determined by qRT-PCR. **p < 0.01; (**D)** MCF-7 cells were treated with or without erastin for 24 h and cultured in sugar-free medium for 24 h. Western blot showing the protein levels of CYCs, HO-1 and SOD2 in each group. (**E)** Densitometric analysis of the gel blot normalized by its own control. **p < 0.01, ***p < 0.001 versus control group; ^##^*p* < 0.01, ^###^*p* < 0.001 versus erastin group. (**F)**MCF-7 cells were transfected with lentivirus containing FoxO3a shRNA, the cells were treated with or without erastin(16 μM) and cultured in normal or sugar-free medium for 24 h, then the levels of antioxidant protein GPX4/SOD2/HO-1/CYCs/FoxO3a were detected by Western blot assay. Densitometric analysis of the gel blot normalized by its own control for **(G)** CYCs, **(H)** HO-1 and **(I)** SOD2. **p < 0.01, ***p < 0.001. Data are expressed as mean ± SEM, n = 3.

The antioxidant genes are important downstream target genes of FoxO3a. Among them, FoxO3a targets mitochondrial superoxide dismutase (SOD) and cytochrome *c* (CYCs). We used Western blot and quantitative real-time PCR analysis to investigate the expression of FoxO3a-regulated anti-oxidative enzymes SOD2, CYCs and HO-1. The results showed that erastin or glucose deprivation stimulated the mRNA and protein expression of CYCs, and glucose deprivation enhanced the expression of CYCs stimulated by erastin; the mRNA and protein levels of HO-1 increased after the erastin treatment (Fig. 3A–E). We observed much higher HO-1 mRNA and protein expression in erastin-treated cells, but there was no significant change under energy stress conditions. In addition, the mRNA and protein expression levels of SOD2 barely changed in erastin-treated cells or under energy stress conditions (Fig. 3B–E). These results suggested that CYCs with high expression may be resistant to ferroptosis.

To further investigate the regulatory relationship between FoxO3a and antioxidant genes, MCF-7 cells were transfected with a FoxO3a lentivirus to down-regulate FoxO3a. As expected, the western blotting results showed that the protein expression level of CYCs was significantly reduced, while the protein expression level of HO-1 and SOD2 had not changed after FoxO3a knockout in erastin-treated cells under energy stress conditions (Fig. 3F–I). These results suggest that activated FoxO3a regulates the expression of the antioxidant gene CYCs.

### 3.4 Energy stress inhibits erastin-induced mitochondrial dysfunction by inducing FoxO3a

**Fig. 4:**
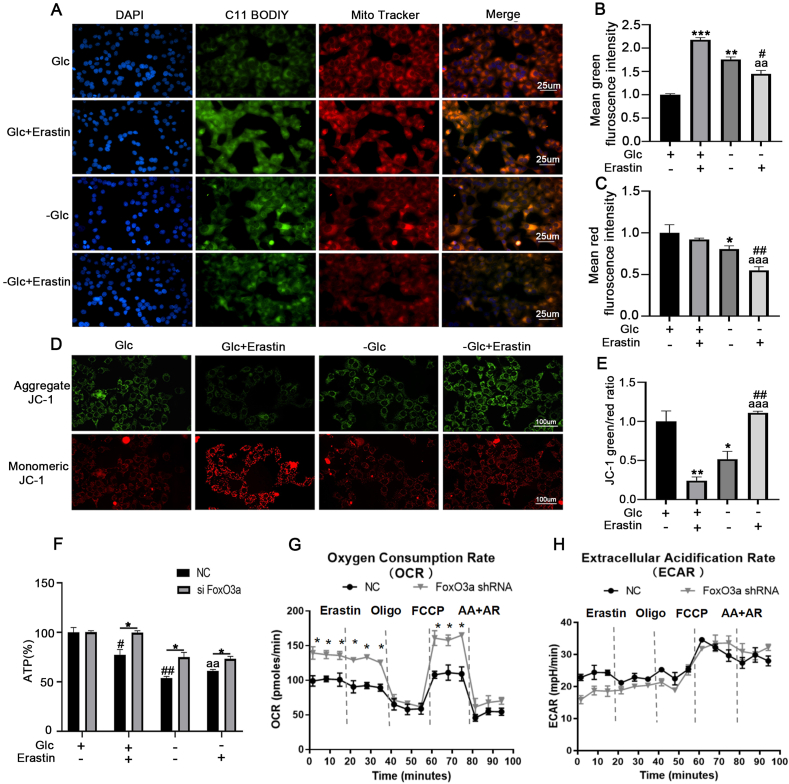
**Energy stress inhibits erastin-induced mitochondrial dysfunction. (A)** Co-localization of oxidized lipid (Green) and mitochondria (Red). MCF-7 cells were treated as indicated for 24 h, and then cells were co-stained with C11-BODIPY581/591 and MitoTracker.**(B)**Mean fluorescence intensity of lipid ROS **(C)** mitochondria in MCF-7 cells after different treatments, **p＜0.01,***p＜0.001 versus the Glc group,^#^*p*＜0.05 versus the -Glc group,^aa^*p*＜0.01 versus the erastin group. Oxidized C11-BODIPY581/591 indicating lipid ROS and mitochondrial signals were imaged by fluorescent microscope **(D/E)** JC-1 fluorescence was observed by fluorescence microscope to detect MMP. Red fluorescence represents the mitochondrial aggregate form of JC-1. Green fluorescence represents the monomeric form of JC-1.*p＜0.05，**p＜0.01 versus control group, ^##^*p*＜0.01 versus the -Glc group, ^aaa^*p*＜0.001 versus the erastin group.**(F)** ATP levels in after transfecting FoxO3a shRNA of MCF-7 cells. ATP production was analyzed via commercial kit. **(G**–**H)** MCF-7 cells were treated with erastin (16 μM) for 6 h before monitoring OCR and ECAR by Seahorse XFp assay for 95 min. OCR and ECAR detected before and after sequential treatment with erastin,ATP synthase inhibitor oligomycin, the uncoupler FCCP, the respiratory inhibitors rotenone and antimycin A at indicated times. *p < 0.01 versus NC group,^#^*p*＜0.05 versus the -Glc group,^aa^*p*＜0.01 versus the Glc + erastin group. Data are expressed as mean ± SEM, n = 3.

Mitochondria are the main source of ROS within mammalian cells, playing an important role in erastin or cysteine deprivation-induced ferroptosis. To determine whether mitochondria play a role in energy stress resistance to ferroptosis, we used the mito-tracker and C11-BODIPY581/591 probes to detect the mitochondrial conditions and lipid peroxidation of cells that were treated with erastin under glucose deprivation conditions. Pictures of mito-tracker-stained cells revealed that glucose deprivation altered mitochondrial appearance from a filamentous network to more punctate structures induced by erastin; the total area of red fluorescence was reduced (Fig. 4A–C), suggesting that glucose deprivation causes a contraction of the mitochondrial network. Immunofluorescence was used to check the co-localization of lipid ROS (green) with mitochondria (red). We found the green fluorescence first appeared in a distribution that significantly co-localized with mitochondria in erastin-treated MCF-7 cells while glucose deprivation significantly decreased the level of lipid ROS accumulation compared with erastin-treated cells (Fig. 4A–B). These results suggests that energy stress inhibited lipid ROS accumulation in mitochondrial reduced cells.

We further used the JC-1 probes to investigate mitochondrial membrane potential (MMP), our results indicate that erastin induced MMP hyperpolarization**,** while glucose deprivation blocked erastin -induced MMP hyperpolarization (Fig. 4D–E). ATP is produced by mitochondria through the utilization of the proton electrochemical gradient potential across the mitochondrial membrane. When mitochondria are reduced or depleted, the levels of ATP will decrease. We further examined ATP levels in cells using commercial kit, and found that ATP levels were clearly reduced in the glucose-deprivation or/and erastin-treated groups, whereas FoxO3a knockdown reversed the decline levels of ATP in MCF-7 cells induced by erastin or/and glucose-deprivation (Fig. 4F), indicating that FoxO3a is responsible for the inhibition of mitochondrial activity. To validate the role of FoxO3a in glucose deprivation -induced energy metabolism, cells were treated with erastin under glucose deprivation conditions. Interestingly, knock-down of FoxO3a appeared to enhance basal oxygen consumption, both under normal conditions and erastin administration after 6 h of glucose deprivation (Fig. 4G), implying that FoxO3a inhibits mitochondrial oxidation. On the other hand, knock-down of FoxO3a did not affect ECAR (Fig. 4H), indicating that FoxO3a did not interfere with the glycolytic machinery in MCF-7 cells.

**Fig. 5:**
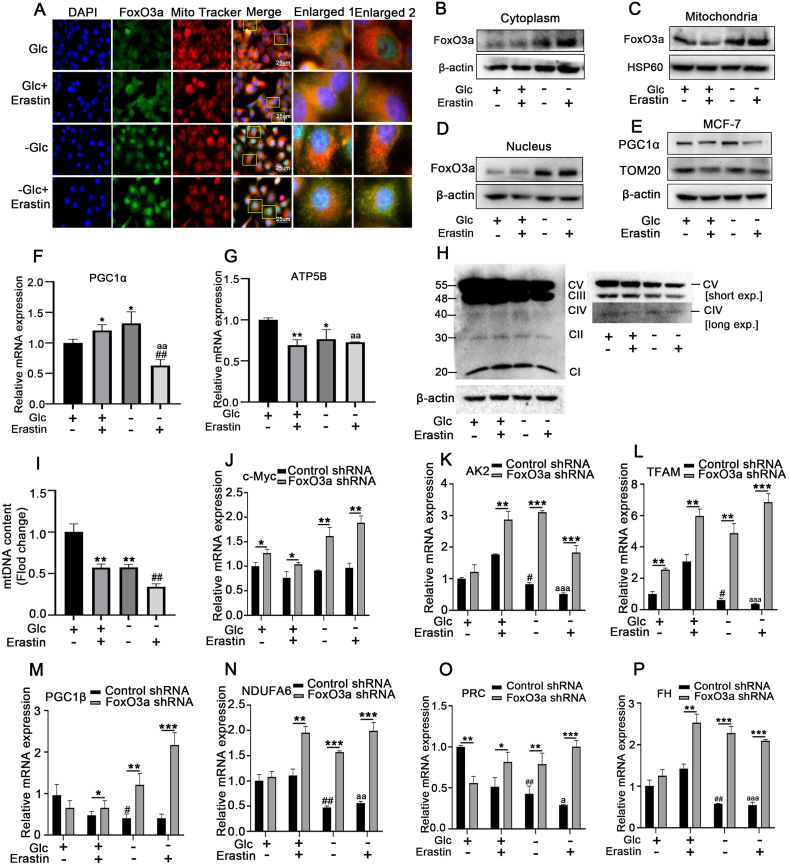
Energy stress inhibits mitochondrial gene expression by inducing FoxO3a **(A)** MCF-7 cells were treated as indicated for 24 h, immunofluorescence colocalization detection in MCF-7 cells using FoxO3a antibody (green) and Mito-tracker (red). The cell nuclei were stained with DAPI (blue),the protein levels of FoxO3a in isolated **(B)** cytoplasm, **(C)** mitochondria and **(D)** nucleus were determined by western-blot, **(E)** the expressions of PGC1α and TOM20 were measured by western-blot. The mRNA levels of PGC1α(**F**) and ATP5B(**G**) were determined by qRT-PCR.*p < 0.05,**p < 0.01 versus the Glc group,^##^*p* < 0.01 versus the -Glc group,^aa^*p* < 0.01 versus the Glc group. **(H)** Total cell lysates were used to detect oxidative phosphorylation (OXPHOS) protein complexes by immunoblotting.**(I)** The mtDNA copy number was measured in each group by qRT-PCR.MCF-7 cells were transfected with lentivirus containing FoxO3a shRNA, the cells were treated with or without erastin(16 μM) for 24 h, the mRNA levels of **(J)**c-Myc, **(K)**AK2, **(L)**TFAM, **(M)**PGC1β, **(N)** NDUFA6, **(O)** PRC and **(P)** FH were measured by qRT-PCR. *p < 0.05, **p < 0.01, ***p < 0.005 versus the control shRNA group. ^#^*p* < 0.05, ^##^*p* < 0.01, ^###^*p* < 0.001 versus the Glc group of control shRNA group. ^**a**^*p* < 0.05, ^aa^*p* < 0.01, ^aaa^*p* < 0.001 versus the Glc + Erastin group of control shRNA. Data are expressed as mean ± SEM, n = 5.

To investigate whether the role of FoxO3a in the regulation of mitochondrial activity confers resistance to erastin-induced ferroptosis. We used immunofluorescence co-localization techniques to detect the expression of FoxO3a in the mitochondria of MCF-7 cells, and immunofluorescence staining showed that FoxO3a expression in both cytoplasm and mitochondria was obviously increased after treatment with erastin or/and glucose-deprivation; the co-immunofluorescence of mitochondria (red) and FoxO3a (green) was found in the control and the erastin-treated cells. Glucose deprivation reduced the number of mitochondria, and a pronounced green fluorescence can be observed in the nucleus under glucose-deprivation conditions, which indicates that a large amount of FoxO3a is activated in the nucleus (Fig. 5A). We further measured the expression of FoxO3a in isolated mitochondria, nucleus and cytoplasm, and we found glucose deprivation induced a significant increase in FoxO3a expression in the mitochondria, nucleus and cytoplasm, and the expression of FoxO3a in the cytoplasm was further enhanced by treatment with erastin(Fig. 5B–D). We next investigated whether FoxO3a activation under energy stress would change the levels of mitochondrial proteins, mtDNA copy number and mitochondrial mass. The result of Western blot showed that glucose deprivation slight decreased of TOM20 expression compared with normal conditions but caused a strong reduction of PGC1α and TOM20 expression in the presence of the erastin(Fig. 5E). We investigated the expression level of PGC1α and its target genes ATP5B by qRT-PCR. Our results showed that glucose depletion inhibited PGC1α and its downstream genes ATP5B expression in erastin-treated MCF-7 cells. However，either glucose depletion or erastin treatment alone stimulated PGC1α mRNA expression while inhibited ATP5B mRNA expression(Fig. 5F–G). Moreover, glucose deprivation decreased the amounts of mitochondrial respiratory complexes in MCF-7 cells treated with erastin, which is indicative of decreased mitochondrial oxidative phosphorylation (OXPHOS) activity(Fig. 5H).We further determined the mtDNA copy by qPCR. Our results indicated that glucose deprivation caused about 50% reduction in the mtDNA copy number and this effect was enhanced after treatment with erastin(Fig. 5I). Above results confirmed that glucose deprivation lowered the mitochondrial mass under erastin treatment as evidenced by downregulation of PGC1α/ATP5B and TOM20.

It has been reported that FoxO3a activation inhibited nuclear-encoded genes with mitochondrial function. To further determine the inhibitory effect of FoxO3a on mitochondria-associated genes, we transfected MCF-7 cells with lentivirus containing FoxO3a shRNA to down-regulate FoxO3a. The qRT-PCR results showed that glucose deprivation significantly inhibited the expression levels of mitochondria-associated gene, including adenylate kinase 2(AK2), mitochondrial transcription factors B2 (TFAM), peroxisome proliferator-activated receptor gamma co-activator- 1β (PGC1β) and PGC-related1 (PRC), fumarate hydratase (FH), and NADH: ubiquinone oxidoreductase subunit A6 (NDUFA6) (Fig. 5J-P). The silencing of FoxO3a significantly increased the expression of all mitochondria-related genes in the erastin or/and glucose deprivation group, and knockdown of FoxO3a induced TFAM expression while reducing PRC expression compared to the control group (Fig. 5L, O). Our data strongly suggest that glucose starvation inhibits erastin-induced ferroptosis at least partly through FoxO3a-mediated inhibition of mitochondrial gene expression.

### 3.5 Trifluoperazine, a potent FoxO3a agonist, protects rats from focal cerebral ischemic/reperfusion injury

**Fig. 6:**
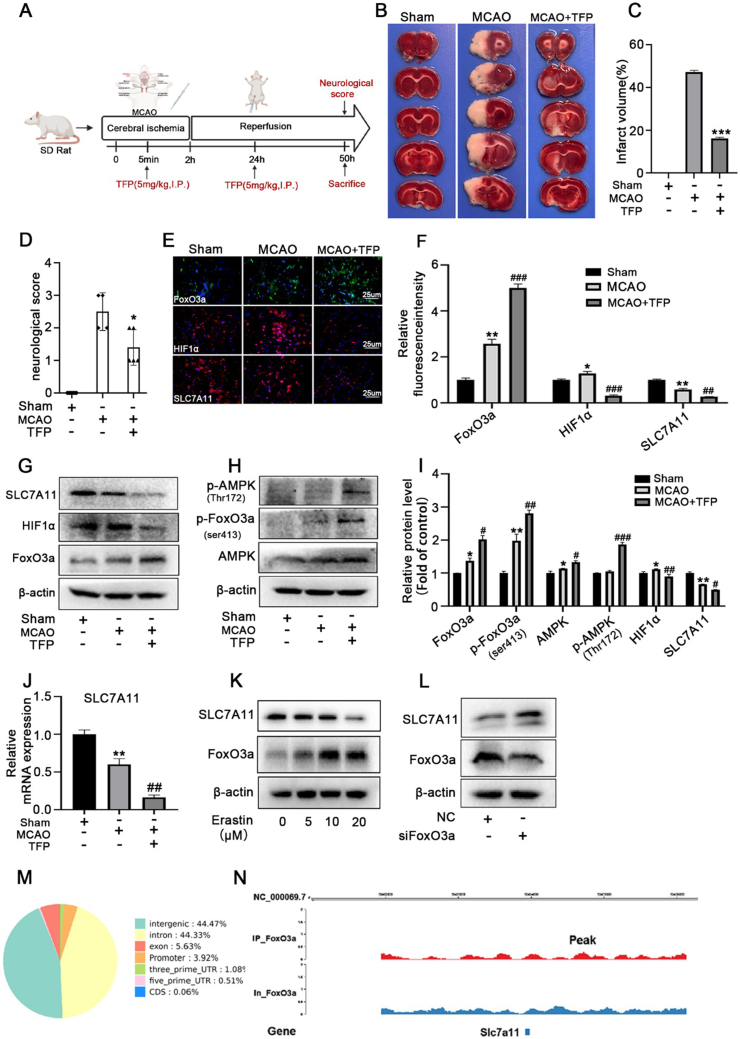
TFP plays a protective effect on CIR injury in rats through AMPK/FoxO3a/Hif1α pathway **(A)**Experimental design of this study. For the sham group rats, only their blood vessels were exposed, and no embolization coils were introduced. Rats in the trifluoperazine groups were treated medically after MCAO surgery for 5 min and repeated 24 h after first injection. Neurological score and Collection of brain were evaluated at 48 h of reperfusion, and tissue samples were subsequently tested. **(B)**The representative TTC-stained brain slices from each group, (**C)** Quantitative analysis of brain infarct volume. **(D)** Effect of TFP on neurological scores. **(E/F)** Immunofluorescence staining results of the penumbral brain tissue in each group. DAPI (blue), FoxO3a (green), Hif1α and SLC7A11 (red). **(G/H/I)**The protein expression of SLC7A11, FoxO3a, *p*-FoxO3a(ser413), AMPK and *p*-AMPK(Thr172) in the penumbral brain tissue were detected by western blotting analysis **(J)**The mRNA expression of SLC7A11 in the penumbral brain tissue were detected by qRT-PCR analysis.**(K)** BV-2 cells were treated with different concentration of erastin for 24 h then the expression of FoxO3a/SLC7A11 were measured by western blotting analysis. **(L)** BV-2 cells were transfected with FoxO3a siRNA for 36 h, then the expression of SLC7A11 were measured by western blotting analysis. *p < 0.05, ***p < 0.001 versus control group; ^#^*p* < 0.05, ^##^*p* < 0.01, ^###^*p* < 0.001 versus MCAO group**.(M)** Statistics of distribution of FoxO3a-binding sites in the mouse genome. **(N)** FoxO3a binding profile containing the gene of SLC7A11 in BV-2 cells. Data are expressed as mean ± SEM, n = 5.

To investigate the FoxO3a function in ferroptosis-associated pathological conditions *in vivo*, we identified FoxO3a activator trifluoperazine (TFP), a FDA-approved antipsychotic agent, and used it in rat exposed to cerebral ischemic/reperfusion injury (CIR). Rat are subjected to transient middle cerebral artery occlusion (MCAO) for 2 h, followed by reperfusion. Trifluoperazine (5.0 mg kg^−1^) was injected intraperitoneally 5 min after the induction of ischemia. Neurological scores and infarct volumes were evaluated at 48 h of reperfusion(Fig. 6A). 2,3,5-triphenyltetrazolium chloride (TTC) staining assays showed that TFP treatment for 24 h at the onset of brain ischemia significantly reduced infarct volume after the induction of CIR in rats (Fig. 6B and C). Moreover, TFP improved the neurological behaviors (Fig. 6D). Given that AMPK directly phosphorylated FoxO3a at Ser413 and led to an enhanced FoxO3a-dependent transcription, we found TFP significantly increases the expressions of AMPK/*p*-FoxO3a(Ser413)/FoxO3a induced by CIR (Fig. 6E–I). These results indicated that TFP exerts a protective effect on CIR injury at least partly through AMPK/FoxO3a activation.

FoxO3a activation blocks the increase in ROS levels and inhibits HIF-1α mRNA after exposure to hypoxia. Consistent with before, we found TFP treatment induced FoxO3a activation while abolishing HIF-1α induction during CIR in rats (Fig. 6E–I). Moreover, histological analysis demonstrated that CIR blocked SLC7A11 expression, and TFP efficiently enhanced CIR-induced downregulation of SLC7A11. This observation was further confirmed with western-blot and qRT-PCR (Fig. 6G–J). To investigate the role of FoxO3a in the regulation of SLC7A11, BV-2 cells were stimulated by erastin in different doses for 24 h. Western blot analysis showed the expression of FoxO3a was increased while SLC7A11 expression was decreased in a dose-dependent manner (Fig. 6K), we transfected BV-2 cells with specific siRNA to inhibit the expression of FoxO3a. Interestingly, knockdown of FoxO3a significantly decreased basal levels of SLC7A11(Fig. 6L). We further conducted ChIP-seq to obtain the chromatin DNA bound by FoxO3a. Among these binding regions, 42.41% were in the intron, 37.96% were in the intergenic of chromosomes, 11.16% were in the exon, 5.37% were in the promoters of these genes, 1.97% and 1.11% were in the 5′ and 3′ untranslated regions (UTRs), respectively. 0.01% were in the CDS of genes (Fig. 3G). What's more, we found there were 2 peaks (peaks 180924 and 180925) annotated in the promoter region of SLC7A11 (Fig. 6M − N). These results indicate that FoxO3a bound to the SLC7A11 promoter inhibits its expression.

### 3.6 Pharmacological activation of FoxO3a by TFP restores the iron alterations, glutamate levels and lipid peroxidation in transient focal brain ischemia in rats

**Fig. 7:**
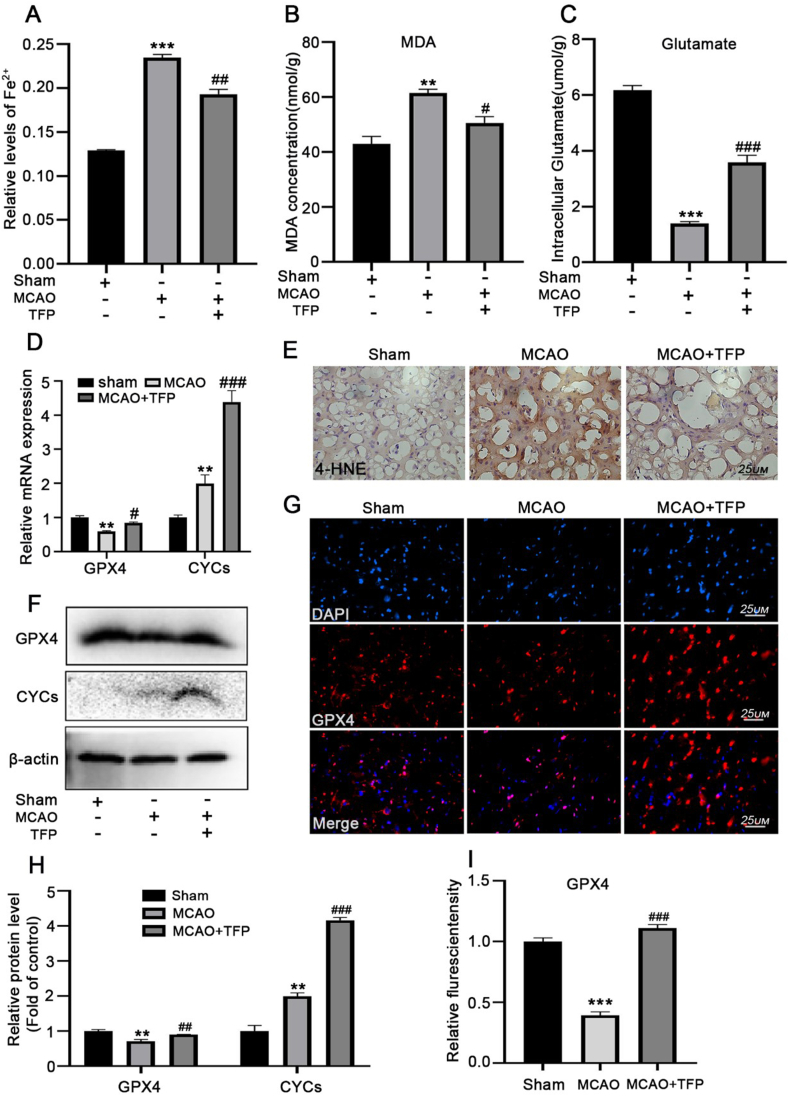
TFP restores the oxidative stress and iron alterations in transient focal brain ischemia in rats. **(A**)Fe^2+^ levels, **(B)**The relative MDA levels and **(C)** the intracellular glutamate levels in the penumbral brain tissue were analyzed via commercial kit. **(D)**The mRNA and protein **(F/H)** levels of GPX4 and CYCs in each group were detected by western blotting and qRT-PCR, respectively. **(E)** Immunohistochemical staining of 4-HNE from rat brain after I/R injury**. (G/I)**Immunofluorescence staining for DAPI (blue) and GPX4 (red) in the penumbral brain tissue. **p < 0.01, ***p < 0.001 versus sham; ^#^*p* < 0.05, ^##^*p* < 0.01, ^###^*p* < 0.001 versus the MCAO group. Data are expressed as mean ± SEM, n = 5.

Deprivation of oxygen and energy triggers an ischemic cascade, and iron overload exaggerates neuronal damage during reperfusion. We showed that TFP reduced the accumulation of Fe^2+^ induced by CIR (Fig. 7A), as indicated by lipid peroxidation analysis using a commercial MDA kit and 4-hydroxy-2-noneal (4-HNE, a lipid peroxidation marker) staining. We have shown that TFP treatment inhibited CIR-induced 4-HNE staining and MDA levels in the penumbral brain tissue (Fig. 7B and E). We also measured the intracellular glutamate levels using a kit, we found the intracellular glutamate concentration was significantly decreased in the penumbral brain tissue, which was reversed after TFP treatment (Fig. 7C). In addition, the low expression of GPX4 indicated enhanced ferroptosis. Our results showed that TFP treatment reversed the decline levels of GPX-4 and enhanced the CYCs expression during CIR (Fig. 7D, F,7G,7H,7I). These results indicated that TFP treatment inhibited CIR-induced neuronal ferroptosis, which potentially relates to an inhibitory effect on lipid peroxidation and glutamate excitotoxicity.

**Fig. 8:**
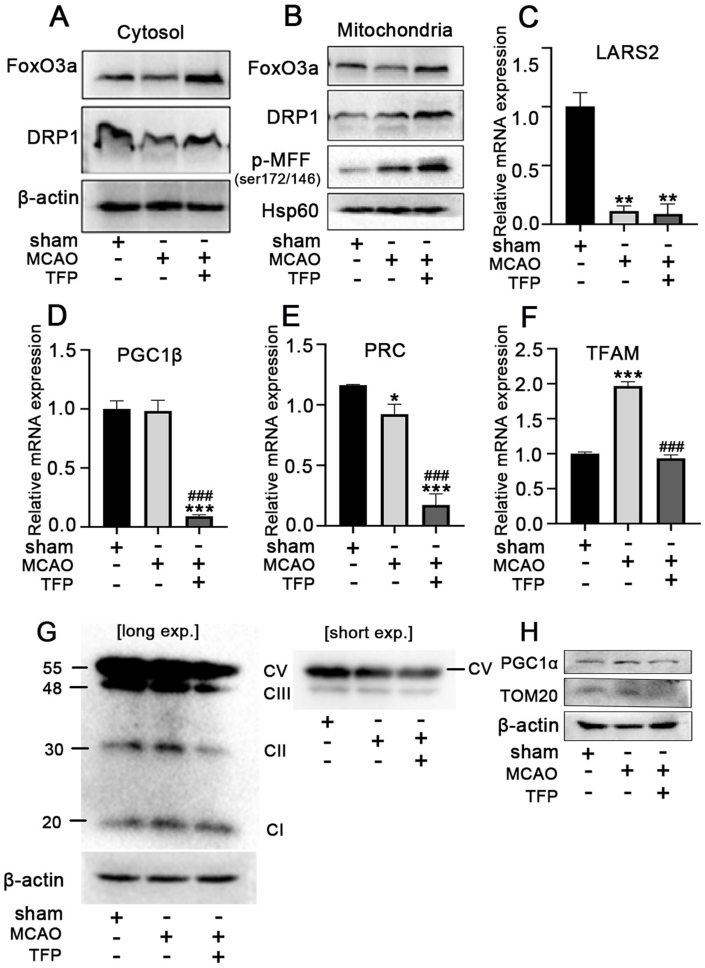
**TFP attenuates CIR injury via mitochondria-associated ferroptosis pathways. (A)** The protein levels of FoxO3a and DRP1 on the cytosol of the penumbral brain tissue was detected by western blotting analysis. **(B)** The protein levels of FoxO3a DRP1and *p*-MFF(ser172/146)on the mitochondria of the penumbral brain tissue was detected by western blotting analysis, qRT-PCR was performed to detect the mRNA expression levels of **(C)** TFAM, **(D)** PRC, **(E)** PGC1β and **(F)** LASR2 in the penumbral brain tissue of rats in each group. **(G)** The whole cell lysate from the penumbral brain tissue were used to detect OXPHOS complexes and **(H)** PGC1α/TOM20 by immunoblotting.**p < 0.01,***p < 0.001 versus the sham; ^###^*p* < 0.001 versus the MCAO group. Data are expressed as mean ± SEM, n = 5.

To further determine whether AMPK/FoxO3a activation promotes mitochondrial fission. Western blotting results displayed that, compared with the CIR group, TFP significantly increased the expression of mitochondrial FoxO3a and Drp1 in the penumbral brain tissue (Fig. 8A and B). Furthermore, qRT-PCR was used to detect the mRNA levels of mitochondria-related genes (LARS2, TFAM, PGC-1β, and PRC) in the penumbral brain tissues of rats in each group. Our results showed that the mRNA levels of LARS2 and PRC were significantly decreased compared with the sham group, while the mRNA expression levels of TFAM were significantly increased in the penumbral brain tissue of rats after CIR. TFP treatment further decreased the CIR-induced downregulation of LARS2 PGC-1β, and PRC while inhibiting CIR-induced TFAM expression (Fig. 8C–F). To investigate whether FoxO3a activation is associated with OXPHOS and PGC1α in response to CIR stress. We investigated OXPHOS complex and PGC1α levels in the penumbral brain tissues. The results showed that total levels of OXPHOS complex-I and II up-regulated in adaptation to CIR, TFP treatment downregulated Complex II, III and IV levels as compared to CIR group (Fig. 8G). We also found the expressions of PGC1α and TOM20 were decreased by TFP treatment in response to CIR stress(Fig. 8H). In effect, these results indicate that TFP modulated the FoxO3a-Drp1-mitochondrial fission pathway and mitochondrial function, which is of great significance for the neuroprotective effect after CIR.

## 4 Discussion

Glucose deprivation can lead to energy stress and markedly increase the intracellular level of ROS [16,17]. Ferroptosis is induced by ROS-mediated lipid damage, which is involved in many human diseases, including cancer, ischemia/reperfusion and traumatic brain injury [18,19]. Mitochondria are the main source of ROS and are also vital for regulating ferroptosis [4]. FoxO3a is involved in the regulation of mitochondrial function and energy homeostasis [20,21]. Previous studies found that mitochondrial dysfunction resulted in insufficient ATP supply and activation of the AMPK/FoxO3a pathway [22,23]. However, whether FoxO3a mediates mitochondrial activity in regulating ferroptosis remains unknown. In this study, we demonstrated that glucose deprivation reduced MMP hyperpolarization, mitochondrial mass and lipid peroxidation and ATP levels, resulting in resistance to ferroptosis induced by erastin. Energy stress stimulated AMPK/FoxO3a signaling leading to the induction of FoxO3a's target genes CYCs and inhibition of mitochondria-related genes and mitochondrial activity as well as lowered the levels of respiratory complexes. Finally, we show that, TFP, a FDA-approved drug exerts a protective effect on CIR injury in rats is related to its inhibition of ferroptosis, and the mechanism might be related to its regulation of AMPK/FoxO3a/HIF-1α/SLC7A11 and mitochondria activity.

AMPK is a critical sensor of cellular energy status that regulates an adaptive response under energy stress. A recent study showed that energy stress activated AMPK and inhibited ACC, resulting in restrained fatty acid synthesis (FAS) and ferroptosis inhibition [6]. Moreover, FoxO3a activity, which is evoked by the AMPK pathway, plays an unique role in energy stress response and ROS regulation. We provide evidence that AMPK phosphorylated FoxO3a at Serine 413 under energy stress, which enhanced FoxO3a-dependent transcription and resisted ferroptosis induced by erastin. Glucose deprivation enhances the gene expression of FoxO3a, which targets CYCs, while HO-1 and SOD2 have little regulation. Mitochondrial CYCs play an anti-oxidative role in the generation and elimination of oxygen (O2) and H2O2 [24]. Our data suggest that FoxO3a might target mitochondrial CYCs, leading to reduced ROS in MEF and MCF-7 cells. Our data demonstrated that FoxO3a deficiency significantly increased these cells' susceptibility to ferroptosis and decreased CYCs expression induced by erastin. As a result, our findings suggest that energy-stress-mediated AMPK activation inhibits ferroptosis via FoxO3a/CYCs-dependent mechanisms.

In this study, knocking down FoxO3a in MCF-7 cells significantly restored ferroptosis sensitivity induced by erastin but not induced by RSL3 under energy stress. Mitochondria promote erastin-induced or cystine-starvation-induced, but not RSL3-induced ferroptosis [4]. This possibility is supported by the fact that FoxO3a had multiple effects on mitochondrial function and regulated energy-stress-mediated inhibitory effects on lipid peroxidation and ferroptosis. Previous studies showed that FoxO3a activation reduces mitochondrial capacity through inhibition of c-Myc and lowers the entry of pyruvate into the tricarboxylic acid (TCA) cycle by induction of PDK4 [8]. Given that TCA cycle is essential for ferroptosis-associated MMP hyperpolarization [4], we subsequently used the JC-1 probe to determine the role of glucose deprivation in ferroptosis-associated MMP hyperpolarization. Our data suggest that glucose deprivation blocks erastin-induced MMP hyperpolarization. Furthermore, the fact that knocking out FoxO3a reversed the decline in ATP levels in MCF-7 cells caused by erectin or/and glucose deprivation, combined with the results generated from seahorse analyzer that knockdown FoxO3a resulted in a significant increase in OCR suggests that FoxO3a is involved in the inhibition of mitochondrial activity.

It has been reported that FoxO3a inhibits mitochondrial gene expression by reducing c-Myc stability [8]. Our data clearly showed that glucose deprivation significantly inhibited the expression levels of mitochondria-associated genes (AK2, TFAM, PGC1β, PRC, FH, and NDUFA6), while silencing of FoxO3a increased expression of all mitochondrial genes and made cells more sensitive to ferroptotic death. These results are in line with the data that mitochondria-mediated ferroptosis might contribute to the antitumor function of FH, and loss of FH function renders cells more resistant to ferroptosis [4]. Further, we show that these results indicated that glucose deprivation activates FoxO3a which also reduced the number of mitochondria and caused a contraction of the mitochondrial network. Consistent with before [8],FoxO3a activation induced by glucose deprivation decreased total levels of OXPHOS complex in erastin treated cells. We measured total levels of PGC1α, an important regulator of mitochondrial biogenesis [25]and mitochondria marker TOM20 in our studies. We found glucose deprivation decreased the total expression of PGC1α and TOM20, indicating energy stress decreased mitochondrial biogenesis in erastin-treated conditions. Consistent with this hypothesis, we identified a FDA-approved antipsychotic agent, trifluoperazine (TFP) was able to activate FoxO3a, inhibited mitochondria-associated genes and OXPHOS complexes as well as mitochondrial proteins in a CIR model. All these observations support the major role of FoxO3a in mitochondria-associated ferroptosis under energy stress.

Ferroptosis is involved in organ ischemia/reperfusion injury, including brain, heart and kidney, and thus represent a potential therapeutic target [[26], [27], [28]]. Previous studies showed that FoxO3a can inhibit HIF1α-dependent gene expression by directly binding to HIF-1α [9]. In particular, ROS derived from mitochondria are required for the accumulation of HIF-1α in hypoxia [11]. HIF1α knockout in brain reduces hypoxic–ischemic damage [29]. Because our findings indicate that energy-stress-mediated FoxO3a activation inhibits ferroptosis via mitochondria-dependent mechanisms, we would like to elucidate the relationship between FoxO3a/HIF-1α activity and mitochondria-associated ferroptosis in a CIR model. TFP may play a neuroprotective role in ischemia stroke through the FoxO3a/HIF-1α pathway, and we further show that triggering this pathway by TFP is associated with reducing 4-HNE staining and MDA level in the penumbral brain tissue. Although previous studies have confirmed TFP's neuroprotective effect against CIR-induced injury [30], to our knowledge, this is the first study to show that FoxO3a can be activated by TFP and inhibit ferroptosis in CIR injuries.

In addition, HIF1α directly binds to the promoter of SLC7A11 and promotes long-lasting glutamate excitotoxicity, conditional knockout of HIF-1α in rat reduced extracellular glutamate in CIR [12]. As expected, following ischemia stroke, the expression of FoxO3a was significantly increased, while the expressions of HIF-1α/SLC7A11 were significantly decreased in TFP -treated group as compared to the control group. Moreover, we found that FoxO3a binds to the promoters of SLC7A11 and reduces CIR-mediated glutamate excitotoxicity through inhibiting the expression of SLC7A11. Many studies provide direct evidence supporting the hypothesis that inhibition of SLC7A11 induces ferroptosis and aggravates ischemia [31,32]. High-expression SLC7A11-cells were more resistant to ferroptosis than low-expression SLC7A11-cells in CIR, we found an inverse correlation between SLC7A11 expression and ferroptosis sensitivity. On the other hand, FoxO3a knockout studies in neural stem cells, revealed that FoxO3a is involved in the regulation of hypoxia-dependent genes [33]. Hence, FoxO3a may take over some of HIF-1α′s function in the adaptation to energy stress conditions.

Previous studies showed that glucose deprivation induced mitochondrial fragmentation in MEFs [34]. Mitochondrial fission and the resulting fragmentation are key signals responsible for triggering the AMPK-FoxO3a signaling pathway [35]. Consistent with previous findings [5], glucose starvation resulted in rapid mitochondrial fragmentation in MCF-7 cells. Fragmented mitochondria are associated with increased production of ROS [36]. Previous studies showed that frataxin significantly enhanced erastin-induced ferroptosis, resulting in dramatic mitochondrial morphological changes, including decreased the number of cristae and enhanced fragmentation [37]. However, another studies showed that STING1 promotes ferroptosis through mitofusin 1/2(MFN1/2)-dependent mitochondrial fusion [38]. Opposing conclusions proposed from different studies may be due to different cell lines and different pathological conditions or diseases. The molecular mechanisms about mitochondrial dynamics and function in ferroptosis deserve further investigation.

It has been reported that AMPK promotes mitochondrial fission through phosphorylating mitochondrial fission factor (MFF), the mitochondrial receptor of Drp1 [39]. Glucose deprivation induces mitochondrial fragmentation, which depends on Drp1 [40]. Mitochondrial fission has been linked to mitochondrial remodeling during FoxO3a-mediated muscle atrophy [41]. We observed induction of the fission regulators DRP1 and *p*-MFF upon FoxO3a activation induced by TFP treatment. Furthermore, TFP treatment inhibited mitochondria-related gene expression in the penumbral brain tissue of rats after the induction of CIR. We proposed that induction of mitochondrial fission regulators and downregulation of mitochondria-related gene expression contributes to the morphological changes following FoxO3a activation, which is responsible for the alterations in mitochondrial activity and resistant to ferroptosis.

In conclusion, our study reveals energy-stress-mediated AMPK/FoxO3a signaling activation inhibited ferroptosis through inhibition of mitochondria-related gene and OXPHOS complexs expression and promotion of mitochondrial CYCs expression, glucose deprivation reduced MMP hyperpolarization, oxygen consumption,mitochondrial mass and lipid peroxidation and ATP levels, resulting in resistance to ferroptosis induced by erastin in MEFs and MCF-7 cells. Our study reveals TFP, a novel FoxO3a activator reduced ferroptosis-associated CIR injuries in rats through repression of HIF-1α/SLC7A11 expression and mitochondrial activity. The regulation of FoxO3 by AMPK may play a crucial role in mitochondrial gene expression that controls energy balance and confers resistance to mitochondria-associated ferroptosis in vitro and *in vivo*.

## Ethics approval and consent to participate

The animal experiments were performed according to internationally followed ethical standards and approved by the research ethics committee of Jiangxi University of Chinese Medicine.

## Availability of data and materials

All data generated or analyzed during this study are included in this published article.

## Declaration of competing interest

All the authors declare no conflicts of interest.

## Data Availability

Data will be made available on request.
