# Supplementary material for: Energy stress modulation of AMPK/FoxO3 signaling inhibits mitochondria-associated ferroptosis
Source: Redox Biol. 2023 May 24;63:102760. doi: 10.1016/j.redox.2023.102760 (PMC10244700; doi:10.1016/j.redox.2023.102760)
Supplement: Multimedia component 1 [file mmc1.docx]

| Gene | Forward Primer(5′-3′) | Reverse Primer(5′-3′) |
| --- | --- | --- |
| *Tfam* | TCTGGAGCAGTATTACGACCC | CTGGCTGGAATCTAGCAGTCT |
| *Pgc1β* | CAGGCGATGGTGCAACTCATA | CAGAGCACGTCTTGAGCCA |
| *Prc* | ATCACCTTCGGGAAATATGGGA | TCTTTCTGACAGACGGATATGCT |
| *Ak2* | GCAGAACCCGAGTATCCTAAAGG | TTCCCAGCATCCATAGTTGCC |
| *Ndufa6* | CGCCAAGCTACTTCTACCGC | TCGGACTTTATCCCGTCCCA |
| *Fh* | GGAGGTGTGACAGAACGCAT | ATCTGCTGCCTTCATTATTGC |
| *Lars2* | CACGGGCGAAAAGCTGACT | TGGGTAAGCATGTTCACAGCC |
| *c-Myc* | GTCAAGAGGCGAACACACAAC | TTGGACGGACAGGATGTATGC |
| *Cycs* | GGGCGAGAGCTATGTAATGCAAG | TACAGCCAAAGCAGCAGCTCA |
| *Sod2* | TGACCACCACCATTGAACTT | CGTCACCGAGGAGAAGTACC |
| *Ho-1* | AGCATGTCCCAGGATTTGTC | ACCAGCAGCTCAGGATGAGT |
| *Slc7a11* | TGGGTGGAACTGCTCGTAAT | AGGATGTAGCGTCCAAATGC |
| *Gpx4* | TCCACCGTGTATGCCTTCTCC | CCTGCTGTATCTGCGCACTGGA |
| *Cytochrome b* | GCGTCCTTGCCCTATTACTATC | CTTACTGGTTGTCCTCCGATTC |
| *Pgc1α* | TCTGAGTCTGTATGGAGTGACAT | CCAAGTCGTTCACATCTAGTTCA |
| *Atp5b* | TGCCCCTGCTACTACGTTTG | TGGCTGAGACAAGAAACGCT |
| *β-actin* | ACCCACACTGTGCCCATCTAC | TCGGTGAGGATCTTCATGAGGTA |

Supplementary Information for

**Energy stress modulation of AMPK/FoxO3 signaling inhibits mitochondria-associated ferroptosis**

Sufang Zhong^a#^，Wenjin Chen^a#^, Bocheng Wang^a#^, Chao Gao^a#^，Xiamin Liu^a^， Yonggui Song^b^, Hui Qi^a^, Hongbing Liu^a^, Tao Wu^a^*, Rikang Wang^a^*, Baodong Chen^a^*

^#^Authors contributed equally to this work.

** Corresponding author.* Dr.Rikang Wang, rkwang@pkuszh.com;

Dr. Baodong Chen, bdchen@pkuszh.com;

Dr.Tao Wu, wutao2002cn@163.com.

**Table S1 Primer for mRNA expression analysis**

Tfam, mitochondrial transcription factors B2 ;Pgc1β,peroxisome proliferator-activated receptor gamma co-activator-1β;Prc,PGC-related1 ; Ak2,adenylate kinase 2;Ndufa6, NADH: ubiquinone oxidoreductase subunit A6 ;Fh,fumarate hydratase ;Lars2,leucyl-tRNA synthetase 2;Cycs,cytochrome c;Sod2,superoxide dismutase;Ho-1,heme oxygenase-1;Slc7a1,cystine-glutamate antiporter;Gpx4,glutathione peroxidase-4;Pgc1α,peroxisome proliferator-activated receptor gamma, coactivator 1 alpha;Atp5b,ATP synthase subunit β, mitochondrial.


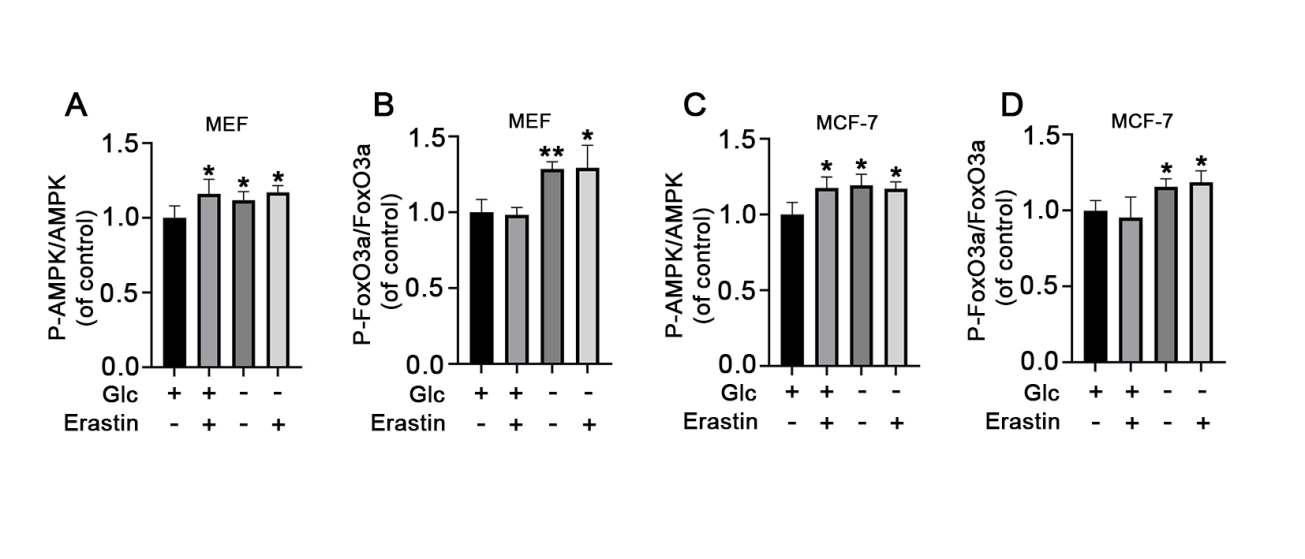


**Figure S1. The ratio between p-AMPK/p-FoxO3a and total protein of AMPK/FoxO3a was calculated by Image J software.** MEF cells or MCF-7 cells were treated with or without erastin (16μM) and cultured in normal or sugar-free medium for 24 h. Quantification of the ratio of p-AMPK to AMPK was measured by western blot in **(A)**MEF cells and in **(C)**MCF-7 cells. Quantification of the ratio of p-FoxO3a to FoxO3a was measured by western blot in**(B)** MEF cells and in **(D)**MCF-7 cells. * *p*＜0.05 , *p***＜0.01 versus the Glc group.
